# Police Discrimination and Depressive Symptoms in African American Women: The Intergenerational Impact of Genetic and Psychological Factors on Blood Pressure Study

**DOI:** 10.1089/heq.2021.0167

**Published:** 2022-07-11

**Authors:** Jolaade Kalinowski, Ryan D. Talbert, Brandy Woods, Aisha Langford, Haile Cole, Veronica Barcelona, Cindy Crusto, Jacquelyn Y. Taylor

**Affiliations:** ^1^ Columbia University School of Nursing and Center for Research on People of Color, New York, New York, USA.; ^2^ Department of Sociology, University of Connecticut, Storrs, Connecticut, USA.; ^3^ Morehouse University School of Medicine, Atlanta, Georgia, USA.; ^4^ Department of Population Health, NYU Langone Health, New York, New York, USA.; ^5^ Department of Anthropology, University of Connecticut, Storrs, Connecticut, USA.; ^6^ Department of Psychiatry, Yale University School of Medicine, New Haven, Connecticut, USA.

**Keywords:** police discrimination, mental health, African Americans, African American women

## Abstract

**Introduction:**

There are significant and pervasive disparities in police violence and discrimination toward African Americans/Blacks. It is possible that these disparities may lead to heightened vulnerability for poor mental health outcomes. The purpose of this study was to ascertain the associations between experiences of police discrimination and depressive symptoms in a community-based sample of African American/Black women.

**Methods:**

We performed a cross-sectional multivariable regression analysis using data from the Intergenerational Impact of Genetic and Psychological Factors on Blood Pressure Study that were collected over a 4-year period from September 2015 to June 2019. Depressive symptoms were assessed using 21 items from the Beck Depression Inventory. Police discrimination was assessed by questionnaires ascertaining experiences of discrimination by police; harassment by police or security guards; and experiences of being unfairly stopped, searched, threatened, or abused by police.

**Results:**

The analytical sample included 214 participants. Nineteen percent of participants indicated that they believed they experienced harassment from security guards/police due to their race/ethnicity. Fourteen percent of participants indicated that they had been unfairly stopped, searched, questioned, or abused by police. Police harassment was associated with higher depressive symptoms by an average of 4.48 (standard error [*SE*]=1.35, *p*<0.001). African American/Black women who were unfairly stopped, searched, or abused by police had higher depressive symptoms by an average of 4.54 (*SE*=1.57, *p*<0.01).

**Conclusion:**

African American/Black women who experienced police discrimination experienced higher prevalence of depressive symptoms. There is an urgent need for reliable population-level data on police mistreatment and interventions at the individual, community, and societal levels.

## Introduction

Racism is a pervasive and systemic public health epidemic^1^ that has detrimental mental and physical health implications.^2–4^ Police discrimination represents a particularly lethal component of institutionalized racism that disproportionately affects African Americans.^5–7^ Recent data suggest that African Americans are at heightened risk for lethal force by police than Whites and 1 in 1000 Black men will be killed by police.^8^ Data for Black women are also especially concerning as they are more likely to be killed by police in comparison with other racial and ethnic groups. The police killings of African Americans such as George Floyd, Breonna Taylor, and countless others have brought considerable public attention to police brutality and racism and how police discrimination may affect health, yet this area remains empirically understudied.

Research suggests that there is a relationship between police discrimination and mental health outcomes. Previous studies show that African Americans are more likely to worry about police brutality than other racial/ethnic groups.^9^ Experiences of worrying may lead to substantial stress and can take a toll on emotional health.^10^ A systematic review on police interactions and mental health of African Americans suggested that there were associations between police interactions and depression as well as other mental illnesses such as post-traumatic stress disorder and anxiety.^11^ This is concerning given that depression tends be more severe and disabling in African Americans^12^ and that there is robust evidence regarding the association of mental health and development of cardiovascular disease and other health concerns.^13,14^

In addition to the nuances involved in understanding the relationship between discrimination and mental/physical health, there may be gender differences with regard to the effects of police brutality on mental health between men and women. For example, literature suggests that Black mothers, in particular, may have the experience of worrying about their children^15^ in addition to other parenting stressors that also disproportionately affect Black mothers.^16,17^ Yet, there is a dearth of research that examine gender differences between men and women.^18^

Despite recent interest in the realm of police discrimination and mental health, there is limited research on how police brutality affects the mental health of African American women. The goal of this study is to evaluate associations between police discrimination and depressive symptoms in a community-based sample of African Americans/Black women.

## Methods

This research was conducted with permission from the Yale University, Columbia University and New York University School of Medicine Institutional Review Boards. The IRB approval information for InterGEN at Yale was 1311012986, and Columbia is AAAS9653. We performed a cross-sectional analysis of data from the Intergenerational Impact of Genetic and Psychological Factors on Blood Pressure Study. Mother and child dyads (*n*=250) were recruited from head start education centers and community-based recruitment in several centers across Connecticut. Inclusion criteria for the study were women aged ≥21 years, African American/Black, fluency in the English language, and absence of cognitive impairment. If there was evidence of cognitive impairment, a follow-up face-to-face Mini-Mental State Examination was conducted. Participants were considered ineligible to participate if there was evidence of a thought disorder, active psychosis, mania, cognitive impairment, or indication that they were incapable of reporting on their and their children's experiences.

Data collection began in September 2015 and continued through June 2019. Psychological measures were collected at each of four study visits, conducted 6 months apart. Study procedures and design methodology have been published previously and can be found elsewhere.^19,20^

Police mistreatment variables were conceptualized using single-item measures from the major experiences of discrimination subscale of the experiences of discrimination scale, which was assessed using the following question: “[Have you] ever been harassed by police or security guards because of [your] race or ethnicity?” We also used a single-item measure from the race-related events scale: “[Have you] ever been unfairly stopped, searched, questioned, physically threatened or abused by the police?” Both items were single-item yes/no measures and captured at time 1.

Depressive symptoms were assessed using 21 items from the Beck Depression Inventory (BDI), a commonly used indicator of mental health with excellent reliability shown among low-income African American medical outpatients.^21^ Respondents answered 21 items that ask about symptoms of sadness, guilt, agitation, sleeplessness, and appetite loss with scores ranging from 0 (or symptom not present) to 3 (or high symptom presence) reflecting the intensity of symptoms. Scores are summed and range from 0 to 44, with higher scores indicating greater depressive symptoms (Cronbach's *α*=0.0.9).

We selected covariates based on previous research regarding depressive symptomatology and police mistreatment.^11^ We controlled for educational attainment since higher education is associated with lower psychological distress.^22^ Educational attainment was categorized, ranging from 1 (less than a high school degree) to 7 (doctorate). We also controlled for categorical household income that ranged from 1 (<$5,000) to 10 ($100,000 or more). Age was examined as a continuous measure. Health insurance status and employment status were coded as dichotomous measures where yes=1.

### Data analysis

For this particular analysis, we examined variables from time point 1 because this represented the most complete wave of data, which led to an analytical sample of 214. The analyses began with the estimation of descriptive statistics for the full sample. The multivariable analyses began with a series of ordinary least squares regressions, but we found that our dependent variable, depressive symptomatology, violated the assumption of normality, and consequently, the models to fit poorly. Furthermore, we ran a Diagnostics for HierArchical Regression Models (DHARMa) test for zero inflation, and found evidence for it.

Thus, using the full sample, we estimated three zero-inflated regression models to examine whether experiences with police mistreatment were associated with depressive symptomatology while accounting for differences in age, education level, employment status, health insurance status, and household income. We treated the nonzero values as continuous and the zero-inflation portion of the model using binary logistic regression. The first model identified associations between harassment by police or security guards because of one's race/ethnicity and depressive symptoms. The second model examined whether unfair stops, searches, threats, or abuse by police was associated with depressive symptoms.

## Results

### Descriptive statistics

In Table 1, we present descriptive statistics for sociodemographic variables. Respondents averaged 31.5 years of age (standard deviation [*SD*]=5.70). Over one-third of respondents had a high school diploma or equivalency (35%). Over 20% earned a household income of <$5,000. Approximately 96% of sample had health insurance, and 70% of people reported being employed.

**Table 1.** Descriptive Statistics for Sociodemographic and Study Variables in the Intergenerational Impact of Genetic and Psychological Factors on Blood Pressure Study, 2015 (*n*=214)

African American women	
Variables	Mean/proportion
Dependent variable	
Depressive symptomology	6.5
(range 0–44, α=0.9)	(7.40)
Police mistreatment (yes=1)	
Police/security guard harassment	0.19
Unfairly stopped/abused by police	0.14
Age (in years)	31.50
(range 21–46)	(5.70)
Educational attainment	
Less than high school	0.06
High school diploma	0.35
Some college, no degree	0.33
Associates degree	0.12
Bachelor's degree	0.10
Master's degree	0.03
Doctorate degree	0.01
Overall mean (SD)	3.0 (1.2)
Income level	
<$5,000	0.22
$5,000 to $9,999	0.14
$10,000 to $14,999	0.12
$15,000 to $19,999	0.08
$20,000 to $24,999	0.08
$25,000 to $34,999	0.13
$35,000 to $49,999	0.12
$50,000 to $74,999	0.06
$75,000 to $99,999	0.03
$100,000 or higher	0.02
Overall mean (SD)	4.2 (2.6)
Health insurance (yes=1)	0.96
Employment status (yes=1)	0.70
Number of children	
None	0.01
1 child	0.26
2 children	0.32
3 children	0.23
4 or more children	0.18
Overall mean (SD)	2.49 (1.5)
Marital status	
Married	0.24
Unmarried	0.75
Other	0.01

SD, standard deviation.

Women reported an average BDI score of 6.50 (*SD*=7.40) with a range of 0–44. Nineteen percent experienced harassment from police or security guards because of their race/ethnicity. Fourteen percent of the sample reported being unfairly stopped, searched, questioned, or abused by the police. With regard to depression, most respondents (74%) exhibited scores in the “normal” range of depressive symptomology, 15% of respondents exhibited scores in the “mild” range, 5% had scores in the “mild” range, and 4% had scores in the moderate range. One participant had a score in the “severe” category, and one participant had a score in the “extreme” category.

### Police harassment and depressive symptomatology

Multivariable regression results are presented in Table 2. There are two types of regression coefficients. The regression coefficients associated with the zero-inflated part of the model are on the logit scale (logit link). This means that large positive values represent larger probabilities of having a zero score on the depression scale. For the conditional part of the model, the coefficients are on the same scale as the outcome variable (identity link), for example, the expected change in depression score for a one unit change in the value of a given continuous predictor, holding all other predictors at a fixed value. In model 1, we present results from a zero-inflated model of depressive symptoms regressed onto police mistreatment.

**Table 2.** Zero-Inflated Models of Depressive Symptomatology Regressed on Police Mistreatment Measures and Controls in the Intergenerational Impact of Genetic and Psychological Factors on Blood Pressure Study, 2015 (*n*=214)

Variables	Depressive symptomatology			
	Model 1		Model 2	
	***b***	SE	***b***	SE
Police mistreatment (yes=1)				
Police/security guard harassment	4.48^***^	(1.35)	—	—
Unfairly stopped/abused by police	—	—	4.54^**^	(1.57)
Covariates				
Age (in years)	−0.08	(0.10)	−0.03	(0.10)
Educational attainment	−0.22	(0.59)	−0.24	(0.60)
Income level	−0.43	(0.31)	−0.45	(0.31)
Health insurance (yes=1)	−0.06	(3.01)	0.57	(3.08)
Employment status (yes=1)	0.00	(1.31)	−0.04	(1.33)

**Zero-inflation component**	**Log odds**	**SE**	**Log odds**	**SE**
Police/security guard harassment	−2.49	(1.43)	—	—
Unfairly stopped/abused by police	—	—	−1.02	(0.70)
Income level	−0.18^*^	(0.08)	−0.18	(0.08)
AICc	1321.80		1333.78	

*Note*: Unstandardized coefficients presented with SEs in parentheses.

^*^ *p*<0.05, ^**^p<0.01, ^***^p<0.001 (two-tailed tests).

AICc, akaike information criterion; SE, standard error.

We examined police/security guard harassment because of one's race/ethnicity in model 1. We found a significant association between police harassment and depressive symptoms such that mothers who reported harassment from police had higher depressive symptoms (*b*=4.48, standard error [*SE*]=1.35, *p*<0.001). Finally, we assessed the association between unfair police stops and depressive symptomatology. African American/Black mothers who were unfairly stopped, searched, or abused by police had more depressive symptoms than those who were not unfairly stopped (*b*=4.54, *SE*=1.57, *p*<0.01).

## Discussion

This study adds to the limited literature on how police brutality and harassment impact the health of African Americans/Blacks and, more specifically, African American/Black women. Our findings demonstrated that African American/Black women who experienced police mistreatment experienced higher prevalence of depressive symptoms. Overall, participants in this study exhibited lower prevalence of depression that has been reported by other African American cohorts such as the Jackson Heart Study, which showed a depression prevalence of 22.3%.^23^

It is important to note that comprehensive systematic data collection concerning police brutality is lacking, and researchers have raised the issue regarding lack of reliable data.^24^ Similarly, there are limited data regarding prevalence of police brutality and harassment.^5^ Our study also demonstrated that police harassment was not uncommon among women enrolled in this study, and that ∼20% of participants indicated that they had been harassed by police or security guards because of their race/ethnicity. Almost 15% of the sample reported having been stopped, searched, questioned, or abused by the police.

Our findings are consistent with other studies that have linked negative police encounters with depression. For example, a previous study examining negative police encounters (e.g., police harassment and police avoidance) in Black men also noted that there were associations between police avoidance and harassment with increased depressive symptoms.^25^ Notably, in a study that examined anticipated youth police interactions and depression in pregnant African American women, pregnant African American women exhibited stress and worry regarding anticipated negative police encounters with their children and that it was associated with depressive symptoms.^15^

There are some limitations to consider when interpreting the results of this study. First, this was a cross-sectional study that examined these measures at one time point. Therefore, we did not examine depression over time. This is an important point to consider because depression incidence and severity are subject to variability over time. Another limitation of this study is that this study only measured whether women had ever had an experience of police discrimination and brutality; we do not have data on the severity or recency of the instance of police discrimination.

In addition, we did not include health comorbidities in our analyses and this may be an important point for future studies to explore. Although there are limitations to consider, there are several strengths that this study offers. These data include detailed measures of stressful interactions with police, which is an understudied area of research. This is one of the few empirical studies to examine police brutality and mental health that adds to the growing body of evidence regarding the adverse effects of police brutality on mental health. Second, we relied on a widely used index of mental health, and sampling coverage spanned an understudied though important population, African American/Black mothers.

## Health Equity Implications

The purpose of this study was to describe the relationship between police mistreatment and depressive symptoms in African American/Black women. There is a concerning longitudinal trend of disproportionate police killings of African Americans in the United States.^26^ The results from this study suggest that there may be a relationship between African American/Black women's beliefs around police aggression on the basis of race and depressive symptomology. As another study suggested, there are “hidden injuries” that can also take a toll on mental health as there is a heightened sense of vulnerability and worrying about police brutality and violence.^9^

The stress and worry about police brutality have been consistently found to implicate the health of African Americans and specifically depression.^27–29^ This study underscores that individuals who believe that they experienced police brutality on the basis of their race may also be even more vulnerable than those who do not. For many African Americans, interactions with the police could equate to death and this pervasive worry is not conducive to ideal physical or mental health outcomes.

There is an urgent need for more systematic and reliably collected data on police brutality and killings. There are several crowdsourced data, largely attributable to the lack of consistent and reliable data, which, in turn, facilitates a lack of accuracy on police brutality data.^24^ Furthermore, the results from this study underscore the need for multiple areas of intervention at the individual, community, and societal levels. Future research should explore how to reliably document police brutality, and how to implement interventions to address the systemic issues that are driving these disparities in police brutality.

## Abbreviations Used

BDI: Beck Depression Inventory
SD: standard deviation
SE: standard error
